# Effect of Different Sedation Regimes on Cognitive Functions in Colonoscopy

**DOI:** 10.5005/jp-journals-10018-1239

**Published:** 2017-09-29

**Authors:** Perihan Ekmekçi, Gulbanu Erkan, Hakan Yilmaz, Baturay K Kazbek, Ulku C Köksoy, Güler Doganay, Doganay Filiz Tüzüner

**Affiliations:** 1 Department of Anesthesiology and Reanimation, Ufuk University, Ankara, Turkey; 2 Department of Gastroenterology, Anesthesiology and Reanimation, Ufuk University, Ankara, Turkey

**Keywords:** Cognitive function, Colonoscopy, Sedation.

## Abstract

**Aim:**

To compare the effects of propofol/remifentanil and meperidine/midazolam on postprocedure cognitive function.

**Materials and methods:**

A total of 100 American Society of Anesthesiologists (ASA) score I to III patients undergoing elective colonoscopy were taken into the study and divided into two groups. Exclusion criteria were patient refusal, mini mental test (MMT) <26, The Amsterdam Preoperative Anxiety and Information Scale (APAIS) >10, advanced cardiopulmonary or psychiatric disease, chronic alcohol abuse, morbid obesity, and known allergy to study drugs. In group MM, 2 mg midazolam and 20 mg meperidine was given intravenously and additional 1 to 2 mg midazolam and 20 mg meperidine (with a maximum total of 5 mg midazolam and 50 mg meperidine) was given when bispectral index (BIS) was >80. In group RP, 100 μg/kg/minute propofol infusion and 1 μg/kg remifentanil bolus was administered and additional 0.5 μg/kg remifentanil bolus was given when BIS was >80. Observer’s Assessment of Alertness/Sedation scale (OAA/S) and Facial Pain Score (FPS) values were recorded. Cognitive function was measured by Trieger Dot Test (TDT) and Digit Symbol Substitution Test (DSST).

**Results:**

The study was concluded with 100 patients. Heart rate was slower and BIS values were lower in group RP throughout the procedure. Blood pressure was lower in group RP without clinical significance. There was no difference concerning recovery time and visual analog scores (VASs). In group MM, TDT scores were higher and DSST scores were lower. Satisfaction was higher in group RP.

**Conclusion:**

Propofol/remifentanil combination is better than meperidine/midazolam combination concerning cognitive function in sedation for colonoscopy.

**Clinical significance:**

The addition of BIS monitorization to evaluate the depth of sedation and the negative effects of midazolam meperidine combination on postprocedural cognitive function.

**How to cite this article:** Ekmekci P, Erkan G, Yilmaz H, Kazbek BK, Koksoy UC, Doganay G, Tüzüner F. Effect of Different Sedation Regimes on Cognitive Functions in Colonoscopy. Euroasian J Hepato-Gastroenterol 2017;7(2):158-162.

## INTRODUCTION

Sedation for lower gastrointestinal system is becoming more frequent and while agents with a fast onset, short half-life, and high safety profiles are chosen, the effects on cognitive functions are frequently disregarded. Cognitive deterioration may become more pronounced in deeper levels of sedation and may have a significant impact on return to daily life. Conscious sedation for colonoscopy is widely preferred by endoscopists,^1-5^ and propofol, midazolam, and opioids alone or in combination are often chosen.

Although anesthesiologists are against sedation in endoscopic procedures provided by staff who are not trained in general anesthesia,^6,7^ debate on who should administer propofol is still going on and is likely to continue for a long time. Fast recovery and return to preprocedure mental function following sedation is an important topic. The ideal agent for sedation should cause minimal cognitive dysfunction in the early postprocedure period and allow rapid return to daily life. Midazolam, which is widely used for sedation in endoscopic procedures, is known to cause cognitive dysfunction.^8^ Propofol alone, on the contrary, has been shown to cause less cognitive deterioration,^9^ however, propofol alone for sedation causes higher costs, deeper sedation, and adverse effects as a result. There is a paucity of randomized studies on cognitive dysfunction following sedation in the literature. Although the effect of endoscopy on cognitive function has been widely evaluated in the literature, the usage of BIS has not been sufficiently investigated. The primary endpoint of this result was to compare the effect of sedation techniques of anesthesiologists and endoscopists on cognitive function, while the secondary endpoint was the comparison of adverse effect incidence and patient and endoscopist satisfaction.

## MATERIALS AND METHODS

Ethical approval for this study conforms to ethical guidelines of Helsinki Declaration (Clinical-trials. gov identifier: NCT02486328). After obtaining written informed consent from each patient, APAIS and MMT were conducted. Consecutive outpatients between 18 and 65 years of age who belonged in ASA I to III risk groups and scheduled for elective diagnostic colonoscopy were included in the study. Exclusion criteria were patient refusal, MMT < 26, APAIS > 10, advanced cardiopulmonary or psychiatric disease, chronic alcohol abuse, morbid obesity, and known allergy to study drugs. One hundred patients who were enrolled into the study were allocated to two groups using closed envelope technique. Study drugs were administered by one trained nurse to all patients who was blinded to the group of the patient. The investigator who conducted the cognitive tests was blinded to the group of the patient.

All patients were given bowel cleaning the night prior to the procedure. Demographic data were recorded and TDT and DSST were given before the test in order to establish a baseline. Intravenous route was established and 100 mg dexketoprofen (Florence, Italy) in 100 mL saline was given 20 minutes and 0.15 mg/kg ondansetron (Istanbul, Turkey) 5 minutes before the procedure as preemptive analgesia. The patients were brought to the endoscopy room and nasal oxygen (2 L/min) was given. Noninvasive blood pressure, peripheral oxygen saturation, electrocardiogram, and BIS (Mansfield, USA) monitorization was applied. The level of sedation was measured using BIS and these values were kept between 60 and 80 by adjusting the drug doses. In group MM, 2 mg midazolam and 20 mg meperidine was applied intravenously, and an additional bolus of 1 to 2 mg midazolam and 20 mg meperidine was given if BIS was >80 with a total maximum dose 5 mg for midazolam and 50 mg for meperidine. In group RP, sedation was abuse, morbid obesity, and known allergy to study drugs. One hundred patients who were enrolled into the study were allocated to two groups using closed envelope technique. Study drugs were administered by one trained nurse to all patients who was blinded to the group of the patient. The investigator who conducted the cognitive tests was blinded to the group of the patient.

All patients were given bowel cleaning the night prior to the procedure. Demographic data were recorded and TDT and DSST were given before the test in order to establish a baseline. Intravenous route was established and 100 mg dexketoprofen (Florence, Italy) in 100 mL saline was given 20 minutes and 0.15 mg/kg ondansetron (Istanbul, Turkey) 5 minutes before the procedure as preemptive analgesia. The patients were brought to the endoscopy room and nasal oxygen (2 L/min) was given. Noninvasive blood pressure, peripheral oxygen saturation, electrocardiogram, and BIS (Mansfield, USA) monitorization was applied. The level of sedation was measured using BIS and these values were kept between 60 and 80 by adjusting the drug doses. In group MM, 2 mg midazolam and 20 mg meperidine was applied intravenously, and an additional bolus of 1 to 2 mg midazolam and 20 mg meperidine was given if BIS was >80 with a total maximum dose 5 mg for midazolam and 50 mg for meperidine. In group RP, sedation was initiated with 100 μg/kg/minute propofol (Bad Homburg, Germany) infusion, 1 μg/kg remifentanil (Italy) bolus, and 0.5 μg/kg remifentanil bolus was added if BIS was >80. Bispectral index and hemodynamic monitorization was continued throughout the procedure and OAA/S) scale and FPS values were recorded. Time to reach OAA/S > 3 was recorded at the end of the procedure and pain was evaluated using VAS score for 30 minutes (1: No pain, 10: Unbearable pain). After the procedure, TDT and DSST were repeated at 5th, 15th, and 30th minute. Complications (hypotension, bradycardia, nausea vomiting, hypoxia, and apnea) were recorded. The patient and the endoscopist were asked to evaluate their satisfaction using a 5-point Likert scale (1: Completely unsatisfied to 5: Completely satisfied). The patients were followed up in the Postanesthesia Care Unit for at least 30 minutes and were discharged when Aldrete score reached 9.

Statistical analyses were carried out using IBM Statistical Package for the Social Sciences for Windows version 21.0, Chicago, USA. Assuming there would be a difference of at least 15% in cognitive score changes, it was calculated that at least 45 patients were needed for each group with 5% type I error and 90% power level. Continuous numerical variables were expressed as mean ± standard deviation and median [minimum-maximum], while qualitative variables were expressed as numbers and percentages. Normal distribution of continuous numerical variables was measured using Shapiro-Wilk’s test. For differences concerning numerical, Mann-Whitney *U* test was used, otherwise, differences concerning categorical variables between groups were evaluated using Chi-square test. Intergroup and intragroup differences concerning the changes in BIS, heart rate, mean arterial pressure, SpO_2_, TDT, and DSST were evaluated using variance analysis in repetitive measurements. Bonferroni test was used for binary comparisons in case a difference was detected. Intragroup comparisons of variables for which parametric test assumptions were not met were carried out using Friedman test. Statistical significance was set at p < 0.05.

## RESULTS

This study was conducted between May and June 2015. Two patients were excluded from group MM due to high APAIS scores and one patient was excluded from group RP due to patient refusal. The study was completed with 50 patients in each group. There were no significant differences concerning demographical data between groups (Table 1). Heart rate was lower in group RP starting from the second minute. Mean arterial pressure values were lower in group RP at 3rd, 10th, and 15th minute, but there was no significant hypotension. No desaturation (SpO_2_ < 95%) was observed. Bispectral index values were significantly lower in group RP starting at the 2nd minute throughout the procedure. The TDT scores were higher in group MM at the 5th (p = 0.001), 15th (p < 0.001), and 30th (p < 0.001) minute (Table 2). The DSST scores were significantly higher in group RP at the 15th (p = 0.04) and 30th (p = 0.004) minute (Table 3). There were 17 patients with a FPS > 3 in group MM, while all FPS scores were 3 and lower in group RP (p < 0.001) (Table 4). There were no statistically significant differences concerning adverse effects between groups. Apnea was observed in three patients in group RP. The time to reach OAA/S > 3 was not different between two groups. Similarly, there was no statistically significant difference in VAS scores between groups (p = 0.802). Patient (p < 0.001) and endoscopist (p = 0.002) satisfaction was significantly higher in group RP (Table 5).

**Table 1:** **Table 1:** Demographical data

		*Group MM (n = 50)*		*Group RP (n = 50)*		*p-value*	
Age (years)		57.1 ± 13.6		53.7 ± 10.4		0.223	
Weight (kg)		71.7 ± 11.7		73.1 ± 15.5		0.656	
Height (cm)		163.0 ± 9.5		164.7 ± 10.7		0.466	
Sex (M/F)		19/31		23/27		0.543	
Baseline MMT		28.2 ± 1.3		28.6 ± 1.6		0.227	

**Table 2:** **Table 2:** Trieger dot test scores

		*Group MM (n = 50)*		*Group RP (n = 50)*		*p-value*	
Baseline		32.3 ± 5.6		32.2 ± 6.2		0.934	
5th min		47.5 ± 10.6		40.4 ± 6.8		0.001	
15th min		44.7 ± 9.2		37.8 ± 5.6		<0.001	
30th min		42 ± 7.9		35.4 ± 6		<0.001	

**Table 3:** **Table 3:** Digit symbol substitution test scores

		*Group MM (n = 50)*		*Group RP (n = 50)*		*p-value*	
Baseline		2.4 ± 1.2		2.5 ± 1.7		0.716	
5th min		1.6 ± 1.2		2 ± 1.6		0.251	
15th min		2.1 ± 1.4		2.8 ± 1.8		0.052	
30th min		2.4 ± 1.4		3.5 ± 2.1		0.004	

**Table 4:** **Table 4:** Facial pain scale and observer’s assessment of alertness/sedation scale

		*Group MM (n = 50)*				*Group RP (n = 50)*					
		*Mean ± SD*		*Median [Min-Max*		*Mean ± SD*		*Median [Min-Max*		*p-value*	
FPS		2.9 ± 1.8		3 [0 - 6]		0.7 ± 1.1		0 [0 - 3]		<0.001	
OAA/S > 3		0.4 ± 1.5		0 [0 - 8]		0.4 ± 1.1		0 [0 - 5]		0.615	
(min)											

**Table 5:** **Table 5:** Patient and endoscopist satisfaction, side effects

		*Group MM (n = 50)*		*Group RP (n = 50)*		*p-value*	
Patient satisfaction		2/1/7/13/27		0/0/0/0/0/50		<0.001	
(1-5)							
Endoscopist		1/1/4/8/36		0/0/0/0/0/50		0.002	
satisfaction (1-5)							
Hypotension		1 (%2)		2 (%5.7)		0.566	
Bradycardia		1 (%2)		3 (%8.6)		0.301	
Nausea vomiting		–		–		–	
Apnea		–		3 (%8.6)		0.066	

## DISCUSSION

In this randomized study, midazolam/meperidine is compared with propofol/remifentanil, and the results demonstrate that propofol/remifentanil combination is superior in preserving cognitive function. Even though propofol can be administered using different techniques, administration via an infusion pump in the absence of anesthesiologists is not common.^10^ A more stable plasma concentration can be obtained and fluctuations observed during intermittent boluses can be avoided when propofol is given as continuous infusion which was the reason infusion was chosen over propofol boluses in our study.^11^

Fast recovery and preservation of cognitive function is also an important subject in endoscopic procedures. Riphaus et al^12^ have compared propofol alone with midazolam plus meperidine in a 100 patient study and reported a faster recovery and less cognitive dysfunction in the propofol group. Padmanabhan et al^8^ have investigated the addition of midazolam or fentanyl to propofol in sedation for colonoscopy and report that propofol alone does not exacerbate the loss of cognitive function but prolongs the procedure and makes it more difficult. As a result, adjuvants in this setting seem beneficial.

In light of literature, primary targets for sedation in colonoscopy are adequate analgesia, hemodynamic stability, swift recovery, and discharge in addition to optimal sedation level. Various drugs have been tried and opioids and benzodiazepines have emerged as drugs of choice. Manolaraki et al^13^ have compared remifentanil with meperidine in sedation for colonoscopy and stated that remifentanil provided better hemodynamic stability, early recovery, and discharge. Remifentanil, which has a very short half-life, has the advantages of fast onset of action, and the possibility of using as an infusion for procedures like sedation.^13,14^ By using a combination of propofol and opioid, we aimed to minimize adverse effects caused by these agents. In this manner opioid adverse effects, such as nausea, vomiting, and desaturation which can be observed in this level of sedation have been avoided. Although apnea was observed in three patients from the propofol/remifentanil group, this was not statistically significant (p > 0.05).

In this study, there was no significant difference concerning postprocedure pain between groups, however, patient and endoscopist satisfaction was higher in the propofol/remifentanil group. Preemptive administration of dexketoprofen and opioid presence in both groups support the similarity between pain scores. Additionally, very low dose midazolam and meperidine boluses were administered in group MM if BIS was >80, while in group RP the infusion rate of propofol/ remifentanil was titrated and thus deep sedation was achieved. This could explain the high patient and endoscopist satisfaction.

Although different doses and combinations of drugs have been investigated for gastrointestinal endoscopies, cognitive function has been neglected.^15,16^ Moerman et al^16^ have compared propofol and remifentanil and reported that remifentanil was superior where cognitive function and hemodynamic effects were concerned. However, propofol dose used in that study was 166 μg/kg/minute, which was one-and-a-half times higher than our dose. Propofol and remifentanil infusion was titrated to achieve BIS values between 60 and 80 and the synergistic effect allowed lower doses of both drugs, thus causing a better cognitive effect. Additionally, the relatively high incidence of hypoventilation in that study can be explained by the absence of BIS and using remifentanil as an infusion. This complication was avoided by using low-dose boluses of remifentanil. Similarly, according to a study by Paspatis et al,^17^ BIS monitorization in endoscopic procedures lowers propofol dose.

According to Watkins et al,^9^ postoperative cognitive dysfunction is defined as significant deterioration in cognitive function as quantified by specific neuropsy-chological tests after a surgical intervention. Cohen et al^18^ have investigated the effects of addition of fentanyl or meperidine to low-dose propofol and midazolam on cognitive dysfunction and reported that there were no significant differences concerning cognitive dysfunction based on DSST, Color-Word test, and trail test. Similarly, Hennessy et al^19^ investigated the effect of midazolam and diazepam on cognitive function in 30 patients undergoing upper gastrointestinal system endoscopy and reported that in equivalent doses midazolam causes more antegrade amnesia. According to the authors, the degree of cognitive impairment is directly proportionate to the dose of mid-azolam and the effect of adjuvants like opioids on cognitive dysfunction is not clear. In a study by Padmanabhan et al,^8^ the effect of adding midazolam and/or fentanyl to propofol has been studied and the authors have stated that midazolam higher than 2 mg is a definitive prognostic factor for cognitive dysfunction.

Although conflicting results can be found in the literature about BIS usage in endoscopic procedures, Park et al^20^ have stated that BIS provides effective and safe sedation for endoscopic procedures in a recent meta-analysis. Various neuropsychologic tests have been used for the evaluation of cognitive function.^21^ Cognitive function has been evaluated using DSST and TDT in this study. The DSST measures attention, visual perception, and motor sufficiency.^22^ The DSST scores were lower in group MM and this supports the notion that benzodiazepines cause cognitive dysfunction. The TDT measures motor ability.^23^ As stated in a study by Takayama et al,^24^ it is a sensitive test for the evaluation of motor function. The TDT results were significantly higher in group MM.

In this study which was conducted in order to evaluate cognitive function following endoscopic procedures, propofol and remifentanil combination has come forward as a good option when cognitive functions are concerned. The fact that CogState battery has not been used can be seen as a limiting factor. Additionally, the exclusion of patients older than 65 can be seen as a limitation. The effect of cognitive function on early recovery should be kept in mind and protocols causing minimal cognitive dysfunction while providing optimal sedoanalgesia levels should be formed.

## CLINICAL SIGNIFICANCE

Different drug regimes can be chosen by anesthesiologists and endoscopists for sedation in endoscopic procedures. Cognitive dysfunction can be observed as an adverse effect as a result of drug and dosage chosen. Although cognitive dysfunction is multifactorial (age, gender, comorbidities), minimal deterioration in cognitive function is desired. This study shows that BIS facilitates dose titration throughout the procedure and causes less cognitive dysfunction and thereby provides easy and effective procedure.
